# Reduced synchronized brain activity in schizophrenia during viewing of comedy movies

**DOI:** 10.1038/s41598-019-48957-w

**Published:** 2019-09-04

**Authors:** Pei-Chi Tu, Tung-Ping Su, Wei-Chen Lin, Wan-Chen Chang, Ya-Mei Bai, Cheng-Ta Li, Fa-Hsuan Lin

**Affiliations:** 1 Department of Medical Research, Taipei Veterans General Hospital, Taipei, 112 Taiwan; 2 Department of Psychiatry, Taipei Veterans General Hospital, Taipei, 112 Taiwan; 3 Institute of Philosophy of Mind and Cognition, National Yang-Ming University, Taipei, Taiwan; 4 Department of Psychiatry, Faculty of Medicine, National Yang-Ming University, Taipei, Taiwan; 5 Department of Psychiatry, Cheng Hsin General Hospital, Taipei, Taiwan; 6 Institute of Brain Science, National Yang-Ming University, Taipei, Taiwan; 7 Department of Medical Biophysics, University of Toronto, Toronto, Canada; 8 Department of Neuroscience and Biomedical Engineering, Aalto University, Espoo, Finland

**Keywords:** Neuroscience, Human behaviour, Schizophrenia

## Abstract

Previous evaluation of brain function in schizophrenia has focused on standard experimental tasks, with cerebral response to natural stimuli less clear. This study employed inter-subject correlation (ISC) analysis to investigate the neural basis of humor processing during free viewing of comedy movies in patients with schizophrenia. We recruited 29 patients diagnosed with schizophrenia and 29 healthy, age- and sex-matched controls. Each participant underwent fMRI scanning during two viewings of three comedy movie clips. The ISC map from each participant pair within each population group and each movie viewing was separately derived. The significance of ISC within a group and between two groups were assessed by bootstrapping. The ISC map from each patient pair were also correlated with the product of Positive and Negative Syndrome Scale (PANSS) rating between the same participant pair in schizophrenia patients. Schizophrenia patients showed significant ISC in bilateral lateraloccipital, bilateral superior frontal, left supramarginal, and right lateralorbiofrontal cortices. Compared with the controls, the schizophrenia group exhibited significantly lower ISC in the left superior temporal sulcus, bilateral supramarginal, and bilateral inferiorparietal cortices. Higher clinical severity (higher total PANSS rating) was associated with lower ISC in the middle frontal and middle temporal regions, and also higher ISC in the visual cortex, inferior temporal gyrus, and anterior cingulate. The findings indicated that patients with schizophrenia are characterized by lower ISC in a frontal parietal network while viewing comedy film clips, which implicated a deficit in the cognitive component of humor processing. The lower synchronization in parts of the frontal parietal network also correlated with symptom severity.

## Introduction

Schizophrenia is a devastating psychiatric disorder accompanied by auditory hallucination, delusion, and cognitive impairment. Functional magnetic resonance imaging (fMRI) has been used to evaluate brain activation in schizophrenics when participants perform standardized cognitive tasks to reveal localized hyper- or hypo-activation^1^. However, the response to natural stimuli in the schizophrenic brain is less clear. Inter-subject correlation (ISC) analysis provides a novel method for the evaluation of brain function in natural situations. The method examines the temporal correlations between brains of participants to estimate the degree of brain synchronization in response to common natural stimuli^2^. ISC analysis on fMRI has been applied to study emotions^3,4^, language^5^, episodic memory^6^, sense of risk perception^7^, and sense of humor^8^. The robustness of the method was also supported by a comparison study^9^, where comparison between ISC and traditional General Linear Model (GLM) analysis was made in five distinct controlled research setups. The results indicated that the data-driven ISC analysis revealed the same foci as the model-based GLM analysis. However, ISC analysis is uniquely useful when no explicit model exists, such as cases of studying brain responses elicited by naturalistic stimuli.

Several pioneering studies have employed ISC analysis to investigate brain function in patients with neuropsychiatric disorders. Hasson, *et al*.^10^ quantified the reliability of the fMRI signals in adults with autism spectrum disorder (ASD) during free viewing of a popular movie under conditions approximating real-life situations. Low inter-subject correlation and high intra-subject correlation among autistic patients were found. Salmi, *et al*.^11^ used ISC analysis for patients with ASD and discovered that the high-functioning patients with ASD failed to synchronize with each other. The patients exhibited lower ISC than the neurotypical controls in brain regions implicated in processing social information, including the insula, posterior and anterior cingulate cortex, caudate nucleus, precuneus, lateral occipital cortex, and supramarginal gyrus. Guo, *et al*.^12^ acquired fMRI for patients with the melancholic subtype of major depressive disorder (MDD) during free viewing of emotionally salient films. They identified a marked disengagement of the ventromedial prefrontal cortex when viewing of a film with negative emotional valence. This effect was significantly correlated with the depression severity. While these studies have provided evidence of the feasibility of the ISC method’s use for neuropsychiatric patients, the synchronization of schizophrenia patients’ brain activity when viewing naturalistic stimuli, to the best of our knowledge, has not been explored.

In this study, we employed ISC analysis to investigate the neural processing of humor during the viewing of comedy movies in patients with schizophrenia. Humor represents a complex higher-order emotional process, and several studies have shown impaired humor appreciation in patients with schizophrenia^13–15^. Affected individuals were shown to display the reduced humor recognition associated with impaired theory-of-mind abilities and diminished medial prefrontal activity in response to jokes requiring the attribution of mental states^16^. The neural basis of humor processing has been investigated by using several experimental modalities, and a review of the functional neuroanatomy of humor indicated that a large set of cortical and subcortical brain areas are activated^17^. In particular, the cognitive components of humor are associated with activation in the language and semantic areas, including the inferior frontal gyrus (Brodmann area 45 (BA 45), BA 46 and BA 47) and temporal pole, and If the stimuli involve theory-of-mind components, the medial prefrontal cortex and temporoparietal junction (BA 22, BA 39 and BA 40) are activated. An emotional component is also found to be involved in humor appreciation and it is primarily associated with increased activity in mesocorticolimbic dopaminergic brain areas (that is, the ventral tegmental area, substantia nigra,nucleus accumbens, ventral striatum and ventral mPFC) comprise the amygdala, nucleus accumbens, and ventral striatum.

Recently, two ISC studies have investigated the neural correlates of humor appreciation under natural conditions when viewing comedy movies. They discovered a consistent pattern of brain synchronization in the healthy participants. In particular, synchronized activity between the medial prefrontal cortex and hippocampus during humor appreciation was found^8^. The ISC at the right frontal pole was associated with experiencing humor and ISC was reduced in several frontal and parietal areas when re-viewing a comedy movie^18^. Comedy movies were used to investigate the sense of humor of patients with schizophrenia In a behavioral study by Tsoi, *et al*.^15^. They discovered that such patients exhibited less sensitive humor detection, but their appreciation of humor was similar to the general population^15^.

Here we used ISC analysis to more specifically elucidate the neural substrates of movie viewing and humor processing in patients with schizophrenia. In this fMRI study, we presented three comedy movie clips (about 5 minutes in duration each) to patients with schizophrenia and control participants. Each movie clip was shown twice. At each brain location, we calculated the ISC values across 406 subject pairs across 29 participants in healthy control and schizophrenia patients separately in each movie clip viewing. The significance of the ISC and the difference of ISC between two population groups was estimated by a non-parametric bootstrap method^19^. Then, we also analyzed the correlation between the ISC value and the product of Positive and Negative Syndrome Scale (PANSS) rating across brain locations for each schizophrenia patient pairs.

Based on previous behavioral evidences of apathy, we hypothesized that schizophrenia patients would show less synchronized brain activity in movie viewing. The difference of synchronized brain activity between the first and the repeated viewing of the same movie clip would also be small in schizophrenia patients. The brain activity in schizophrenia patients may be more dissimilar at areas subserving humor processing than that from healthy controls. The symptoms severity of schizophrenia affects the synchronization of brain activity during movie watching. These hypotheses were explicated tested in this study.

## Results

**Figure 1:**
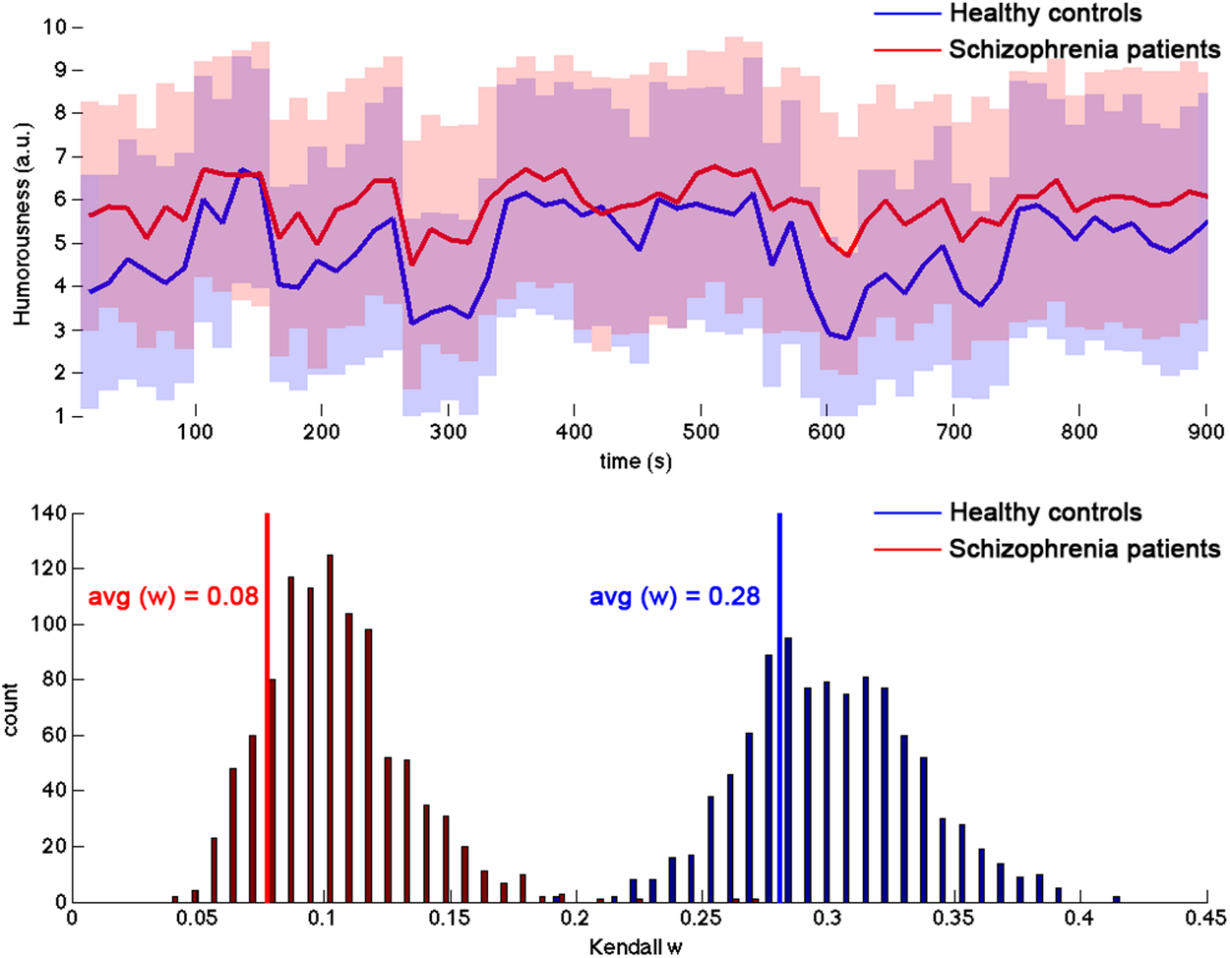
Rating of subjective amusement in participants with schizophrenia and controls. (**a**) Shaded areas represent the standard deviation across participants at each rating interval (15 s). Solid lines represent the average rating across the participants over time. (**b**) Kendall’s W was used to quantify the similarity of a subjective rating within a population group. The histogram shows that the Kendall’s W of the control and schizophrenic groups is significantly different (*p* = 0.001).

The demographic data of all the participants are presented in Table 1. Behaviorally, schizophrenia group showed a trend level of higher subjective amusement than healthy controls (*p* < 0.07, Table 1). We also used Kendall’s W to quantify the similarity of a subjective rating within a population group. A low Kendall’s W indicates that the rating is divergent among the raters in a group. Both controls and schizophrenia show favorable agreement within the population group (control: W = 0.28; *p* < 0.001; schizophrenia: W = 0.08, *p* < 0.001). The histogram shows that the Kendall’s W of the control and schizophrenic groups is significantly different (*p* = 0.001), suggesting that the subjective ratings of the controls have significantly higher agreement than those of the schizophrenia (Fig. 1).

**Table 1:** Participant demographic and clinical status characteristics.

subjects Characteristics	Schizophrenic patients (n = 29)	Healthy controls (n = 29)	t/X^2^	p
Age (years)	38.2 ± 8.2	35.7 ± 9.1	1.11	0.27
Sex (M/F)	19/10	17/12	0.29	0.79
Education level	13.2 ± 2.5	15.1 ± 1.8	−3.24	0.002*
Handedness (R/L)	28/1	27/2	0.35	1.00
Age at onset	23.2 ± 7.3			
Length of illness (years)	15.1 ± 9.3			
PANSS Total	68.4 ± 23.5			
Positive Subscale	15.2 ± 6.5			
Negative Subscale	18.7 ± 7.3			
General Psychopathology Subscale	34.5 ± 11.8			
Subjective rating of amusement	5.88 ± 2.03	4.87 ± 2.11	1.84	0.07

M = Male; F = Female; R = right; L = left; PANSS = Positive and Negative Syndrome Scale for Schizophrenia.

**Figure 2:**
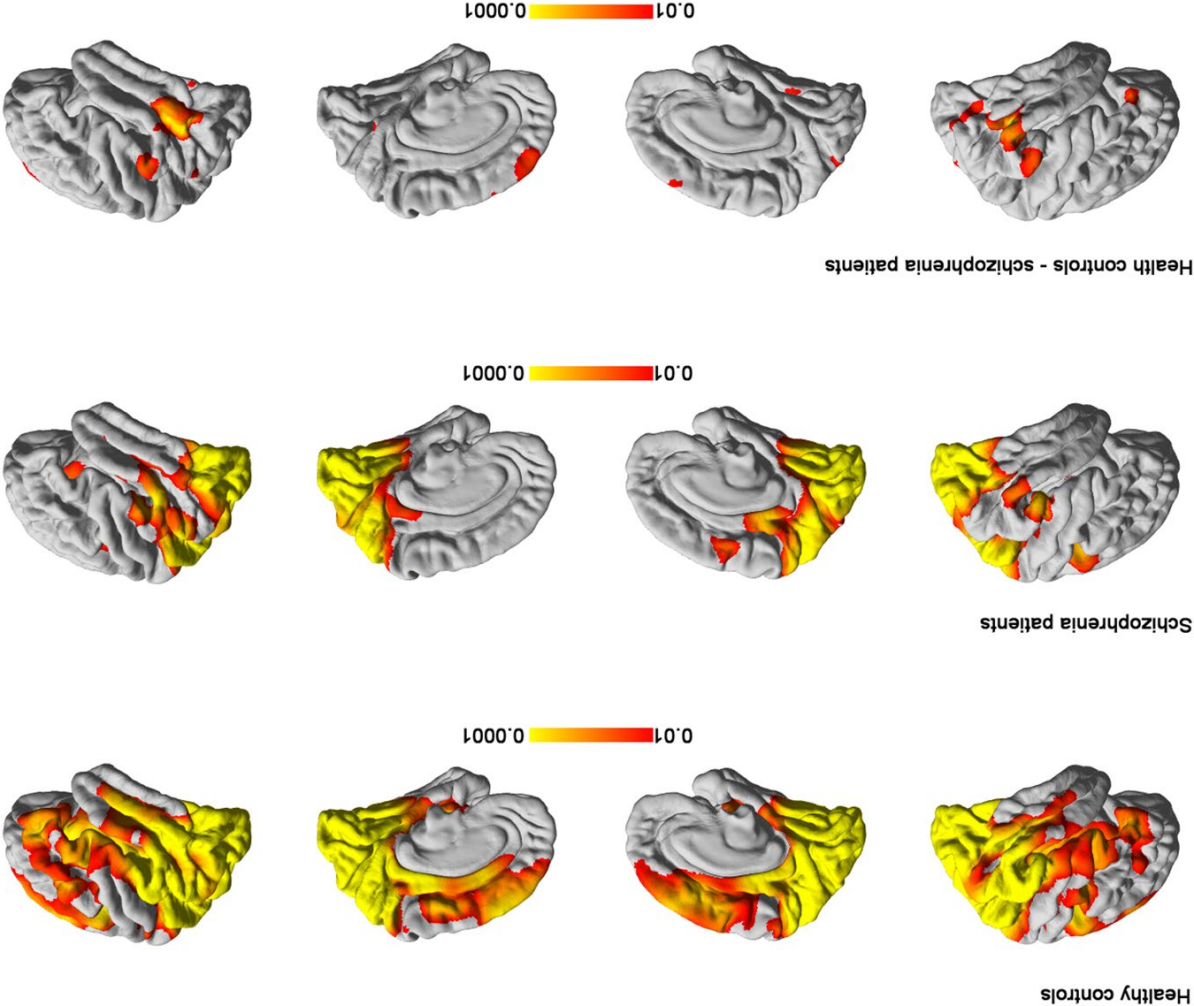
The cortical regions showing statistically significant group-level ISC during the viewing of the film clips in the (**a**) controls and (**b**) participants with schizophrenia; (**c**) The cortical regions showing statistically significant between-group differences. The patients with schizophrenia showed significantly lower ISC in several frontal and parietal regions.

**Figure 3:**
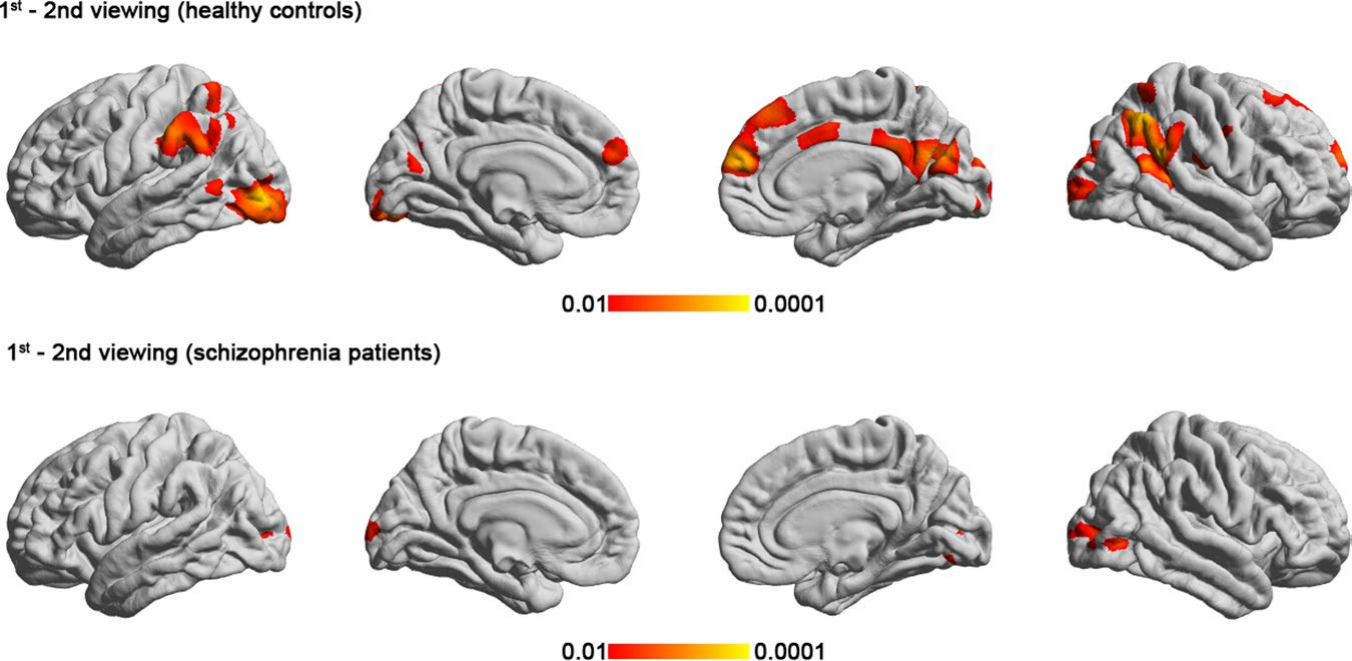
ISC difference between the first and second viewing (2^nd^ -1^st^ viewing) of the comedy movie clips in the (**a**) controls and (**b**) participants with schizophrenia. The control group showed significant reduction in ISC in several frontal and parietal areas during the second viewing. The schizophrenic patients did not have a significant difference in ISC between the first and second viewings

ISC analysis showed that viewing of the comedy movie clips evoked neural synchronization in the primary and secondary visual areas, inferior and middle temporal gyrus, inferior parietal cortex, precuneus, and right middle frontal gyrus in the controls (Fig. 2). Compared with the controls, the patients with schizophrenia showed significantly lower ISC in bilateral lateraloccipital, bilateral superior frontal, left supramarginal, and right lateralorbiofrontal cortices (Fig. 2 and Table 2). Compared with the first viewing, the control group had an extensive significant reduction in ISC in the occipital, frontal, and parietal areas during the second viewing. By contrast, the schizophrenic patients only showed significant difference in ISC between the first and second viewings at some parts of the occipital lobe (Fig. 3 and Table 3).

**Table 2:** The cortical structures showing significant lower inter-subject correlation in patients with schizophrenia.

Max {-log(p)}	Size (mm^2^)	NVtxs	Talairach Coordinates			Structure
			X	Y	Z	
**SZ < HC**						
4.00	1532.02	3808	−51	−47	15.5	L. bankssts
2.64	425.45	1144	−61	−30.9	35	L. supramarginal
2.94	544.57	746	−27.3	−81.1	15.2	L. inferiorparietal
2.68	333.59	476	−46.8	33.3	−5.4	L. parstriangularis
2.36	154.77	444	−41.8	−72	1.4	L. lateraloccipital
4.00	2587.07	5383	46.7	−53.1	12.5	R. inferiorparietal
2.79	402.41	953	60.8	−28.2	38.2	R. supramarginal
2.49	249.26	532	7.1	51.1	35.3	R. superiorfrontal

HC = healthy controls; SZ = schizophrenic patients; L = left; R = right; NVtxs = number of vertices in cluster; bankssts = banks of the superior temporal sulcus.

**Table 3:** The cortical structures showing significant lower inter-subject correlation during 2^nd^ viewing of comedy movies in healthy controls and patients with schizophrenia.

	Max {-log(p)}	Size (mm^2^)	NVtxs	Talairach Coordinates			Structure
				X	Y	Z	
viewing1^st^ < viewing 2^nd^							
**HC**							
	4.00	2453.92	4193	−37.3	−85.0	2.9	L. lateraloccipital
	2.93	1326.71	3559	−59.1	−29.7	37.2	L. supramarginal
	2.57	324.46	924	−29.7	−53.9	59.2	L. superiorparietal
	2.46	230.51	407	−8.6	−64.4	31.1	L. precuneus
	2.48	241.73	309	−7.0	51.9	21.7	L. superiorfrontal
	2.34	179.92	293	−58.2	−58.3	5.5	L. middletemporal
	2.30	140.83	229	−3.5	−73.5	22.1	L. cuneus
	4.00	3179.8	6792	54.4	−42.3	22.9	R. inferiorparietal
	3.66	2390.26	4072	10.8	−61.3	25.6	R. precuneus
	3.54	1432.6	2397	8.8	56.2	18.8	R. superiorfrontal
	2.74	1110.11	1444	24.3	−79.5	24.2	R. superiorparietal
	2.37	395.55	891	6.2	0.4	39.0	R. posteriorcingulate
	2.37	655.94	883	27.9	−92	3.6	R. lateraloccipital
	2.42	413.34	834	27.9	−54.2	61.3	R. superiorparietal
	2.37	486.37	569	10.3	−91.0	−4.6	R. lingual
	2.54	169.36	430	53.1	−19.6	17.9	R. postcentral
**SZ**							
	2.34	164.84	365	−7.0	−100.4	7.7	L. lateraloccipital
	2.20	103	139	−24.2	−86.4	6.8	L. lateraloccipital
	2.58	574.89	767	32.2	−80.3	4.7	L. lateraloccipital
	2.37	397.53	418	21.4	−76.3	−3.9	R. lingual
	2.59	239.41	307	43.9	−70.4	2.1	R. lateraloccipital

HC = healthy controls; SZ = schizophrenic patients; L = left; R = right; NVtxs = number of vertices in cluster.

**Figure 4:**
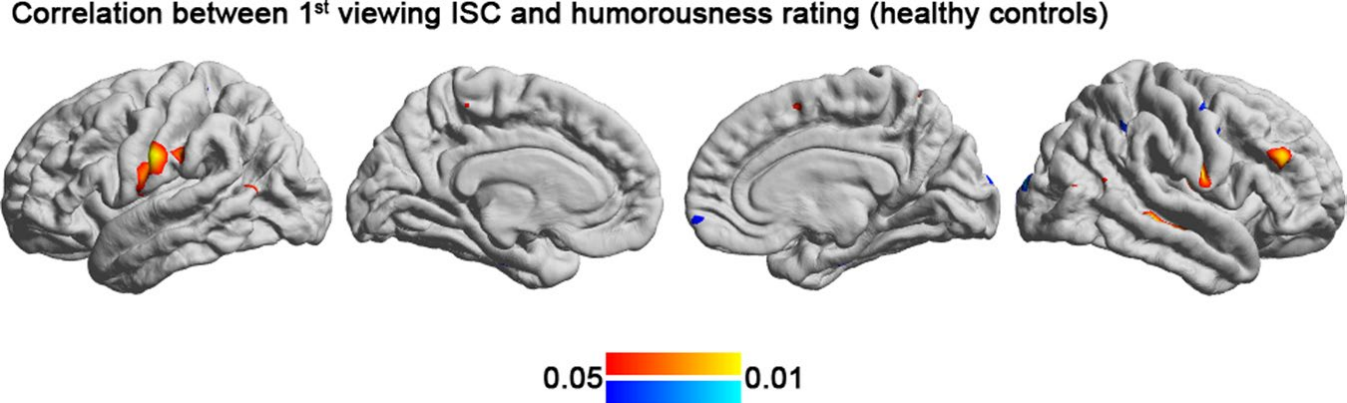
The cortical regions showing significant correlations between the ISC and subjective ratings of humor in the controls.

The correlation analysis between ISC and the subjective ratings of humor demonstrated that the controls exhibited positive correlation of ISC in the right middle frontal gyrus, left inferior parietal cortex, and bilateral postcentral cortex and a negative correlation of that in the right precentral cortex with subjective rating of funniness. However, we did not observe a significant correlation between ISC and subjective rating of humor in the schizophrenia group (Fig. 4, Table 4).

**Table 4:** The cortical structures showing significant correlation between inter-subject correlation and subjective rating of amusement.

	Max T	Size (mm^2^)	NVtxs	Talairach Coordinates			Structure
				X	Y	Z	
**HC**							
Positive	3.85	513.03	1270	−62.1	−8.9	23.5	L. postcentral
	3.41	158.30	438	−40.3	−32.7	35.1	L. supramarginal
	3.16	160.94	358	−55.2	−24.7	29.1	L. supramarginal
	3.71	299.20	663	48.6	−23.5	−8.2	R. middletemporal
	3.85	196.39	458	58.0	−6.5	18.2	R. postcentral
	3.38	136.40	339	51.6	−9.2	16.3	R. postcentral
	3.84	109.39	203	15.4	−51.8	57.9	R. superiorparietal
	3.68	114.39	176	46.1	36.8	23.0	R. rostralmiddlefrontal
negative	−3.28	121.62	238	41.0	−3.8	47.0	R. precentral
**SZ**							
	N.S.	—	—	—	—	—	—

HC = healthy controls; SZ = schizophrenic patients; L = left; R = right; NVtxs = number of vertices in cluster; N.S. = no significant.

**Figure 5:**
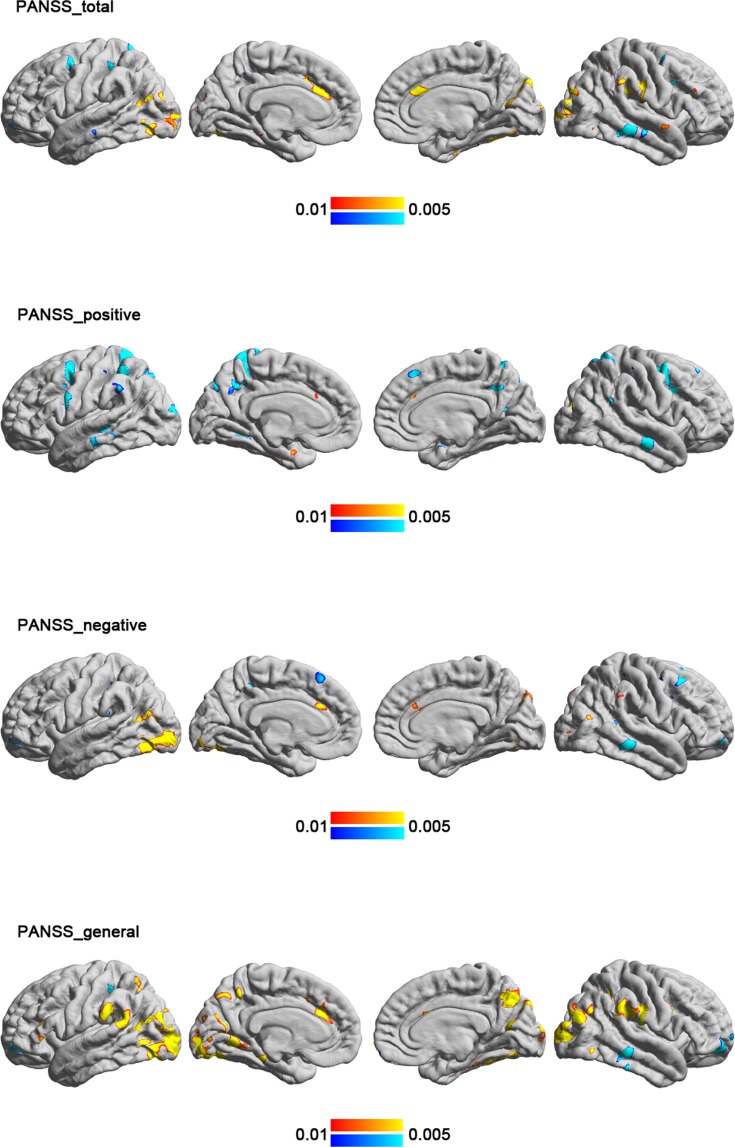
The cortical regions showing a significant correlation with the clinical rating in the schizophrenia group. A higher total PANSS rating was associated with higher ISC in the visual cortex, inferior temporal gyrus, and anterior cingulate as well as lower ISC in middle frontal and middle temporal regions. The correlation between PANSS subscores and ISC were also shown separately.

In the schizophrenia group, a higher total PANSS rating was associated with higher ISC in the visual cortex, inferior temporal gyrus, and anterior cingulate as well as lower ISC in middle frontal and middle temporal regions (Fig. 5 and Table 5). We also explored the correlation between PANSS sub-scores and ISC. The positive symptom scores were correlated with lower ISC in the frontal, parietal, and temporal association cortex, whereas negative symptom scores and general pathology scores were mostly correlated with higher ISC in the occipital and temporal regions (Fig. 5).

**Table 5:** The cortical structures showing significant correlation between inter-subject correlation and total PANSS scores in patients with schizophrenia.

Max T	Size (mm^2^)	NVtxs	Talairach Coordinates			Structure
			X	Y	Z	
**Positive correlation with PANSS (total)**						
3.99	128.59	205	−5.7	26.6	20.2	L. caudalanteriorcingulate
3.99	59.20	126	−33.6	−76.9	−6.0	L. fusiform
3.95	61.84	118	−11.3	15.3	32.6	L. caudalanteriorcingulate
3.98	142.20	299	28.6	−57.8	34.8	R. superiorparietal
3.99	75.16	165	8.3	13.8	27.3	R. caudalanteriorcingulate
3.98	63.11	158	58.7	−16	26.3	R. postcentral
3.98	83.36	119	38.0	−68.3	−7.1	R. fusiform
3.99	81.46	78	11.2	−66.2	14.3	R. pericalcarine
3.98	61.89	56	15.8	−74.8	24.0	R. cuneus
**Negative correlation with PANSS (total)**						
−3.98	50.47	112	−44.4	2.1	45.9	L. precentral
−3.98	69.83	108	−32.7	7.1	54.2	L. caudalmiddlefrontal
−3.98	53.38	65	−30.6	56.8	−7.8	L. lateralorbitofrontal
−3.99	129.97	201	65.2	−15.8	−11.9	R. middletemporal

L = left; R = right; NVtxs = number of vertices in cluster; PANSS = Positive and Negative Syndrome Scale for Schizophrenia.

## Discussion

The present study investigated the neural correlates of humor processing in schizophrenia during viewing of comedy movie clips by using ISC analysis. Behaviorally, the schizophrenic group had a similar level of subjective amusement compared with the control group, but which part of the movie clips they found amusing was significantly less consistent (Fig. 1). In agreement with previous fMRI ISC studies, the viewing of comedy movies caused synchronized hemodynamics in diverse occipital, temporal parietal, and frontal structures in the controls (Fig. 2). Patients with schizophrenia are associated with less synchronized hemodynamics than controls in the inferior parietal cortex, precuneus, superior frontal cortex, and anterior cingulate (Fig. 2). Different from controls, the hemodynamics in the schizophrenia group showed relatively small adaptation effect as there was moderate change in the synchronization of the BOLD signals between the initial and the repeated viewing of the comedy movie clips (Fig. 3). It should be noted that, in the present study, we only presented movie clips of humorous content to patients and healthy controls. Therefore, the synchronized fMRI signals across participants found in the present study can only be attributed to specific complex naturalistic stimuli, rather than “comedy”. While a control condition of non-comedy movie clips is desired to ensure the findings are specifically related to watching humorous movie clips, it is difficult to making two movie clips with the difference only in the presence of “comedy components” is not feasible. Even providing brightness- or complexity-matched movies to participants cannot selectively probe neural substrates underpinning the sense of humor processing. However, we attempted to partially address this issue by correlating between subjective ratings on the degree of humorousness and the inter-subject correlated fMRI signal (Fig. 4). Taking the areas with significant correlation between ISC values and subjective ratings on the degree of humorousness from healthy controls as “humor processing areas” (Fig. 4), we found that (*i*) the ISC values at these areas in schizophrenic patients were insignificant (middle row, Fig. 2), and (*ii*) they were minimally overlapped with areas with significant ISC value difference between healthy controls and schizophrenic patients (bottom row, Fig. 2). This suggested that the humor processing was more diverse across schizophrenic patients.

Although the controls in this study showed significant correlation between subjective feeling of humorousness and ISC in several frontal and parietal regions, the participants in the schizophrenia group did not have any significant correlation between ISC and subjective amusement (Fig. 4). The significant correlation in only healthy controls, but not in patients, suggested areas differently subserving the processing humorous contents between populations. Within the schizophrenia group, greater symptom severity was associated with higher ISC in the visual cortex but lower ISC in the frontal and temporal areas (Fig. 5). Taken together, these results suggested that specific brain areas in patients with schizophrenia have reduced synchronized dynamics when perceiving naturalistic humorous stimuli. And such regional reduction in synchronized brain dynamics is closely related to the severity of clinical assessment.

In this study, we chose the product of PANSS scores to correlate ISC values (Fig. 5). This choice was made by our *a priori* assumption that the degree of disorder presented by the PANSS score can be related to ISC values. One may alternatively postulate that the ISC values can be correlated with the difference of PANSS scores from a subject pair, since that both are measures of “similarity”. Indeed, if there were two subjects both with either very high PANSS scores or with very low PANSS scores, the difference in PANSS score difference between subjects in these two scenarios would be small. However, the difference in PANSS score products between these two scenarios would be large. Thus, choosing the product of PANSS scores allowed us to differentiate symptoms severity of schizophrenia.

One major finding of this study is that the schizophrenia group exhibited significantly lower ISC in several structures, primarily the frontal and parietal areas, while viewing the comedy movie clips. As two ISC studies on ASD and MDD have demonstrated^11,12^, lower ISC is a consistent characteristic of psychiatric disorders and the exact regions activated in the brain may depend on the nature of the stimuli and the disorder. Several possible causes can contribute to the lower ISC in the schizophrenia group: First, the lower ISC may reflect underlying cortical dysfunction in information integration. Frontal and parietal cortices are the association area and have been consistently found to have impaired structure or function in patients with schizophrenia^20,21^; dysfunction in processing natural stimuli may have caused the aberrant hemodynamics among patients and subsequently lower synchronization. Second, lower correspondence in the appreciation of humor may also have contributed to lower ISC in the schizophrenia group. As shown in the behavioral results, significant inconsistency about subjective ratings on the degree of humorousness across schizophrenia patients was identified. The lower correspondence in the subjective feeling of amusement may have contributed to the lower level of synchronized brain activity. Third, lower ISC may reflect a deficit in the processing of humor in the participants with schizophrenia. Although the schizophrenia group exhibited a similar level of subjective amusement behaviorally, they may not have understood the humor in the same manner as the controls. An fMRI study using relatively short sentences and punchlines^22^ discovered that patients with schizophrenia had attenuated BOLD signals in several frontal areas—including the left dorsomedial middle and superior frontal gyri (BA 8/9) and the interhemispheric dorsal anterior cingulate cortex (BA 24)—during the various stages of humor processing. Given the previous theory about the cognitive and emotional components of humor, our findings of lower ISC in the fronto-parietal areas support that schizophrenia patients have the deficit of cognitive processing of humor because of the anatomical overlap.

The schizophrenia group did not exhibit a significant correlation between ISC and subjective perception of humor. By contrast, several areas in the frontal and parietal regions were correlated with subjective feeling of humorousness in the controls and may play a principal role in the processing of humor (Fig. 4). The frontal component involved the right middle frontal gyrus. Previous ISC studies have also identified a correlation between subjective rating and frontal ISC at the right frontal pole^23^ and the left superior frontal gyrus^8^. The role of frontal cortex in humor processing was also supported by previous task fMRI studies that have demonstrated activation of the middle frontal gyrus in humor processing^24,25^. Several parietal structures, including the left inferior parietal, right superior parietal and bilateral postcentral gyrus, were correlated with subjective feeling of humorousness in the controls. The superior and inferior parietal lobes play a crucial role in attention, and clips with more subjective amusement may require more attention and induce higher ISC in these parietal regions. The lack of correlation between the ISC in these fronto-parietal regions and the subjective rating in the schizophrenia group may provide further evidence for the dysfunction of these areas in the processing of humorous stimuli by people with schizophrenia.

The present study also revealed that the schizophrenia group failed to exhibit lower ISC during the second viewing of the comedy film clips, unlike the controls. We interpreted this result as a dysfunction of habituation, which is the decrease in response to a stimulus following repeated exposure with no meaningful consequence^26^. Previous fMRI studies have demonstrated the effect of “adaptation:” a decrement in fMRI response during the second presentation of task stimuli. One study investigated fMRI habituation and discovered reduced habituation in the right anterior hippocampus of people with schizophrenia in response to repeated display of fearful faces^27^. Another study repeatedly displayed a neutral face or neutral object and successive different neutral faces and neutral objects to participants and found that patients with schizophrenia showed reduced habituation of the hippocampus and visual cortex^28^. The present study is the first ISC analysis to demonstrate that patients with schizophrenia failed not only to synchronize brain activity across patients but also to show decreased synchronization during repetitive viewing of movie clips with salient emotional and cognitive components.

This study also explored the correlation between PANSS rating and ISC within the schizophrenia group. We found that the association patterns varied across symptoms’ subscales. The positive symptoms were mostly correlated with a lower ISC in the frontal, parietal, and temporal association cortex, whereas negative symptoms and general pathology were mostly correlated with a higher ISC in the occipital and temporal regions, potentially related to visual processing. We suggest that the dysfunction in the frontal and parietal association cortex in more severe patients may cause the cortical activity more sensory driven. The findings are in accordance with previous resting-state fMRI connectivity studies, which found hyper-connectivity in the sensorimpotor cortex and hypo-connectivity in the prefrontal cortex. These dys-connectivities were found correlated with symptoms severity in schizophrenic patients^29–31^ or adults at elevated clinical risk^32^.

A limitation of this study was that the participants with schizophrenia were receiving various dosages of antipsychotics and antidepressants. The effect of antipsychotics on ISC is unclear and caused a possible confound that may have contributed to the lower ISC findings in this study. Another limitation was that we focused on the cortical areas by using a surface-based method and we were unable to detected potential ISC changes in subcortical areas.

In this study, we used GLM to calculate *t* statistics to evaluate the significance of the correlation ISC values to subjective rating on the degree of humorousness (Fig. 3) or the product of the PANSS scores (Fig. 5). A recent study compared methods of parametric and non-parametric approaches, including permutation and bootstrap, in ISC analysis^33^. With the group size of 20, their numerical simulations found that *t*-test provided higher statistical power at the cost of larger false positive rate. Additionally, *t*-test performed very similar to a few variants of permutation and bootstrap procedures^33^. Considering the computational cost of permutation and bootstrap procedures and similar performance, we chose to use *t* statistics to assess the significance of the correlation between ISC and behavior measures.

Data in this study were pre-processed following the strategy reported in our previously study^23^. Since the volume images were registered into cortical surfaces space, the spatial smoothing can be performed after the surface registration and on the 2D surface, rather than on the 3D volume. However, during registering a brain volume to cortical surfaces of a common coordinate in order to allow for subsequent inter-subject correlation analysis, a 3D spatial smoothing has been employed^34^. Therefore, the difference of two different smoothing procedures may not be significantly different.

## Conclusions

In conclusion, we found that patients with schizophrenia are characterized by lower synchronized brain activity in the frontal and parietal association cortex, lack an association between the degree of synchronized brain activity and subjective amusement rating, and lack habituation in the repeated viewing of movie clips. The reduced synchronization in parts of the frontal parietal network was also correlated with symptom severity. The findings suggest neural substrates related to the deficit in the cognitive component of humor processing in patients with schizophrenia. While we only used comedy movie clips in this study, analyzing synchronized brain activity across patients elicited by complex and naturalistic stimuli and contrasted that with controls can be a useful protocol to study high-level cognitive and emotional dysfunction in other psychiatric disorders.

## Materials and Method

### Participants

We recruited 29 patients diagnosed with schizophrenia and 29 healthy, age-, handedness-, and sex-matched controls. The schizophrenia group comprised outpatients from Taipei Veterans General Hospital in Taiwan that had received a diagnosis of schizophrenia (Table 1). The control participants were recruited through an advertisement. An experienced psychiatrist used the MINI to screen and exclude the candidates with major psychiatric illnesses. In addition, candidates with a history of first-degree relatives with axis-I disorders, including schizophrneia, major depressive disorder and bipolar disorder, were excluded.

The diagnoses of the schizophrenia group were confirmed through structured clinical interviews based on the *Diagnostic and Statistical Manual of Mental Disorders* (the 4^th^ ed.; DSM-IV; American Psychiatric Association, 1994^35^. The clinical status of the patients was characterized by using the Positive and Negative Syndrome Scale (PANSS^36^), and the patients had been receiving treatment with various atypical antipsychotics, antidepressants, and mood stabilizers before participating in this experiment (detailed in Supplementary Table 1). Additionally, before being enrolled in this study, the participants in the schizophrenia group were screened by using the Mini-International Neuropsychiatric Inventory Plus^37^.

The criteria for exclusion from the schizophrenia group were a history of head injury that resulted in a sustained loss of consciousness and cognitive sequelae, neurological illness, or any disorder affecting the cerebral metabolism.

All the procedures were approved by the Institutional Review Board of Taipei Veterans General Hospital, and all the participants provided written informed consent after being apprised of the experimental procedures. A statement confirming that all experiments were performed in accordance with relevant guidelines and regulations.

### Experimental design

The participants watched three movie clips (approximately 5 minutes in duration) twice and without the sound during fMRI acquisition. The movie clips were taken from the comedy movies “The Circus” and “City Lights,” directed by Charles Chaplin and produced by Charles Chaplin Productions in 1928 and 1931, respectively. None of the participants watched either movie before. The participants were instructed to watch the movie clips as they would watch films on television or at the cinema. An angled mirror in front of the participant’s eyes reflected the movie image, which was projected onto a translucent screen in the MRI bore behind the participant’s head. The computer program Presentation (Neurobehavioral Systems, Inc.) was used for visual stimuli delivery. Immediately after the fMRI session, the same three movie clips were re-shown to the participants and the participants were asked to rate the degree of experienced humorousness on a 10-point Likert scale, once every 15 seconds. A rating of 1 corresponded to no humor experienced and a rating of 10 corresponded to a high degree of humor experienced.

### Image acquisition

Images were acquired using a 3T MRI (Discovery MR750, General Electric, USA). Head stabilization was achieved using a cushion. All the participants wore earplugs (29-dB rating) to attenuate the acoustic noise during the MRI scans. Automated shimming procedures were performed and scout images were first obtained. The functional images were collected using a gradient-echo *T*_2_*-weighted sequence (TR/TE/flip angle = 2500 ms/30 ms/90°). Forty-three contiguous horizontal slices, parallel to the inter-commissural plane (voxel size: 3.5 × 3.5 × 3.5 mm^3^) were acquired in an interleaved order. High-resolution structural images were acquired in sagittal planes by using a sequence (TR = 2530 ms, TE = 3 ms, echo spacing = 7.25 ms, flip angle = 7°) with isotropic 1-mm resolution (field-of-view = 256 mm × 256 mm).

### Behavior rating analysis

In addition to comparing the means of the subjective ratings, we used Kendall’s W to assess if the subjective ratings on the degree of humorousness were consistent among the raters in a population group^18^. Specifically, a significantly larger than 0 Kendall’s W suggests that the ratings of the raters in a population group are consistent. To assess if the subjective ratings exhibit difference between groups, we used a bootstrap approach to sample the raters from a population group with replacements and iteratively calculated Kendall’s W 1,000 times. The significance of the difference between the population groups was quantified by counting the number of the occurrences of bootstrap samples from one population group with more extreme Kendall’s W than the Kendall’s W from the other group.

### ISC analysis

The EPI data were first preprocessed by using motion correction, slice-timing correction, and spatial smoothing (with a 10-mm full-width-half-maximum Gaussian kernel). Each brain volume was then spatially co-registered to a template (the “fsaverage” subject in FreeSurfer, version 5.1.0, http://surfer.nmr.mgh.harvard.edu). After surface registration, each hemisphere had 10,242 vertices. The inter-subject correlated fMRI time courses were analyzed using the procedures reported in our previous ISC study^18^. Specifically, the first 10 seconds of data were discarded to achieve the steady state of magnetizations. Pearson’s correlation coefficients were then calculated to quantify the similarity of the fMRI time courses between participants during the movie watching after the removal of the temporal confounds modeled by the zeroth-, first-, second-order polynomials and sinusoidal fluctuations in periods of 2, 1, and 2/3 run durations. The voxel-wise temporal correlation between every pair of participants was calculated at each brain location. These correlation coefficients were Fisher *Z*-transformed^38^ and then averaged across participant pairs, resulting an ISC map. We generated 12 ISC maps for two groups (patients/controls) × 3 movies × 2 viewings.

### Statistical analysis on ISC

The statistical inferences in the ISC maps were derived by using non-parametric bootstrap test^19^. Specifically, participants within the same group were sampled randomly with replacement to calculate inter-subject correlation coefficients across subject-pairs at each brain location. In each bootstrap step, we calculated the median of correlation coefficients across 406 subject pairs (from 29 subjects). The null distribution of the median of correlation coefficients was estimated by shifting the distribution of the median of correlation coefficients across 10,000 bootstrap samples by its mean^19^. The null distribution of the median was estimated separately for each population group and movie clips. The p-value was estimated by the number of the occurrence of the median in bootstrap samples higher than the median in experiment samples. The significant ISC clusters with a size larger than 100 mm^2^ on the cortical surface were automatically found by the *mri_surfcluster* program in FreeSurfer (http://surfer.nmr.mgh.harvard.edu).

### Correlating fMRI ISC with clinical scores and subjective ratings

We used linear regression to reveal the brain areas showing a significant relationship between the fMRI ISC and PANSS scores. Specifically, the ISC values at each brain location across the participant pairs were regressed against the products of the PANSS scores from the matched participant pairs. In other words, the correlation was performed between 406 fMRI ISC values and 406 products of PANSS scores across all pairs from 29 participants in each population. Importantly, we did not average the correlation maps across participant. The statistical inferences were drawn by calculating the *t* statistics from the regression coefficients, which were converted to *p* values after controlling the FDR (*q = *0.05) in multiple comparisons.

To reveal the brain areas with a significant correlation between the fMRI ISC and dynamic subjective ratings, we first used spline interpolation to align the subjective ratings on the degree of humorousness over time to the onset timing of each fMRI volume acquisition. We then calculated the inner product of the two subjective rating time courses from all the participant pairs. The fMRI ISC values at each brain location across the participant pairs were regressed against the inner product of the subjective rating time courses from the matched participant pairs. Again, the statistical inferences were drawn by calculating the t statistics from the regression coefficients. The p values associated with the t statistics were calculated after controlling the FDR (q = 0.05) in multiple comparisons.

## Supplementary information

Supplementary Table 1

## Data Availability

The datasets generated during and/or analyzed during the current study are not publicly available due to the privacy law in Taiwan, but are available from the corresponding author on reasonable request.
