# Supplementary material for: Reduced synchronized brain activity in schizophrenia during viewing of comedy movies
Source: Sci Rep. 2019 Sep 4;9:12738. doi: 10.1038/s41598-019-48957-w (PMC6726596; doi:10.1038/s41598-019-48957-w)
Supplement: Supplementary file 1 — Supplementary Table 1 [file 41598_2019_48957_MOESM1_ESM.docx]

**Title: Reduced synchronized brain activity in schizophrenia during viewing of comedy movies**

Pei-Chi Tu^1,2,3,4^, Tung-Ping Su^2,4,5,6^ , Wei-Chen Lin^2,4,6^, Wan-Chen Chang^1,2^, Ya-Mei Bai^2,4^, Cheng-Ta Li^2,4,6^, Fa-Hsuan Lin^7,8,*^

^1^Department of Medical Research, Taipei Veterans General Hospital, Taipei 112, Taiwan

^2^Department of Psychiatry, Taipei Veterans General Hospital, Taipei 112, Taiwan

^3^Institute of Philosophy of Mind and Cognition, National Yang-Ming University, Taipei, Taiwan

^4^Department of Psychiatry, Faculty of Medicine, National Yang-Ming University, Taipei, Taiwan

^5^Department of Psychiatry, Cheng Hsin General Hospital, Taipei, Taiwan

^6^Institute of Brain Science, National Yang-Ming University, Taipei, Taiwan

^7^Department of Medical Biophysics, University of Toronto, Toronto, Canada

^8^Department of Neuroscience and Biomedical Engineering, Aalto University, Espoo, Finland

^*^Corresponding author: Fa-Hsuan Lin, Ph.D.

Department of Medical Biophysics, University of Toronto, Toronto, Canada.

2075 Bayview Ave, Room M6-607

Toronto, ON, Canada M4N 3M5

E-mail: fhlin@sri.utoronto.ca

Tel: +1 416-480-6100 ext. 85477

Supplementary Table 1 The detailed psychotropics used in patients with SZ in this study

| Medication | SZ (N=29) |
| --- | --- |
| antipsychotics | n=29 |
| Aripiprazole | 2 |
| Clozapine | 1 |
| Haldol | 1 |
| Lodopin | 1 |
| Paliperidone | 8 |
| Quetiapine | 1 |
| Risperidone | 2 |
| Zyprexa | 1 |
| Clozapine + Amisulpride | 3 |
| Clozapine + Aripiprazole | 1 |
| Olanzapine + Paliperidone | 1 |
| Paliperidone + Clozapine | 1 |
| Quetiapine + Aripiprazole | 1 |
| Risperidone + Aripiprazole | 1 |
| Risperidone + Etumine | 2 |
| Aripiprazole + Quetiapine + Risperidone | 1 |
| Clozapine + Paliperidone + Fluanxol | 1 |
| antidepressant | n=11 |
| Cymbalta | 1 |
| Lexapro | 1 |
| Prozac | 1 |
| Seroxat | 1 |
| Valdoxan | 1 |
| Wellbutrin | 1 |
| Zoloft | 3 |
| Pinsaun + Wellbutrin | 1 |
| Valdoxan + Sinequan | 1 |
| mood stabilizers | n=18 |
| Anxiedin | 5 |
| Flupine | 2 |
| Rivotril | 2 |
| Vaproic Acid | 4 |
| Xanax | 1 |
| Anxiedin + Rivotril | 1 |
| Rivotril + Frisium | 1 |
| Vaproic Acid + Rivotril | 2 |
